# 血液病患者细菌性血流感染病原菌分布及耐药特点单中心数据分析

**DOI:** 10.3760/cma.j.cn121090-20240603-00201

**Published:** 2024-10

**Authors:** 梦婷 车, 超盟 王, 海芳 孔, 惠 刘, 丽娟 李, 嘉 宋, 化泉 王, 玉红 吴, 国锦 王, 晶 关, 文 瞿, 莉民 邢, 鸿 刘, 晓明 王, 志东 胡, 宗鸿 邵, 蓉 付

**Affiliations:** 1 天津医科大学总医院血液科，天津 300052 Department of Hematology, Tianjin Medical University General Hospital, Tianjin 300052, China; 2 天津医科大学总医院检验科，天津 300052 Department of Clinical Laboratory, Tianjin Medical University General Hospital, Tianjin 300052, China

**Keywords:** 血流感染, 细菌分布, 耐药性, 血液系统疾病, Bloodstream infections, Bacteria distribution, Drug resistance, Hematological diseases

## Abstract

**目的:**

分析血液病患者细菌性血流感染的病原菌分布和耐药情况，为临床经验性抗感染治疗提供病原学数据依据。

**方法:**

对天津医科大学总医院血液科2016年1月至2022年12月经血培养确诊为细菌性血流感染的血液病患者的一般临床信息、病原菌及药物敏感试验结果进行回顾性分析。

**结果:**

共纳入498例细菌性血流感染患者，共发生619例次感染，分离出639株细菌。其中86.9％为恶性血液病患者，76.7％处于粒细胞缺乏期，65.9％存在导管留置，32.6％出现降钙素原升高，10.7％发生血流动力学改变。常见感染部位为呼吸道（60.6％）、口腔黏膜及皮肤软组织（17.3％）、腹腔（14.5％）。639株细菌中革兰阴性（G^−^）菌占79.0％，革兰阳性（G^+^）菌占21.0％，前5位病原菌依次为肺炎克雷伯菌（22.5％）、大肠埃希菌（20.8％）、铜绿假单胞菌（15.0％）、屎肠球菌（5.5％）、嗜麦芽窄食单胞菌（5.0％）。G^+^球菌检出率在2020–2022年呈上升趋势。肺炎克雷伯菌对喹诺酮类和头孢菌素类药物的耐药率在2016–2018年间有所上升，但在2019年后呈下降趋势。铜绿假单胞菌对碳青霉烯类药物的耐药率约为20.0％，2019年耐药率峰值为35.7％。耐甲氧西林凝固酶阴性葡萄球菌（MRCNS）对甲氧西林的耐药率为60.0％，检测到1例耐利奈唑胺的MRCNS。

**结论:**

本中心细菌性血流感染以G^−^杆菌为主，但G^+^球菌在2020–2022年呈上升趋势，应警惕碳青霉烯类耐药铜绿假单胞菌和肺炎克雷伯菌。

## Abstract (en)

**Objective:**

To analyze the distribution and drug resistance of pathogens of bacterial bloodstream infection in patients with hematological diseases in the Department of Hematology of Tianjin Medical University General Hospital, and to provide etiological data for clinical empirical anti-infection treatment.

**Methods:**

A retrospective analysis was conducted on the general clinical information, pathogenic bacteria and drug susceptibility test results of patients with hematological diseases diagnosed with bacterial bloodstream infection by menstrual blood culture in our center from January 2016 to December 2022.

**Results:**

Patients included 498 inpatients, with a total of 639 bacterial strains. Among the patients, 86.9% patients had malignancies, and 76.7% had agranulocytosis. Symptoms of concurrent infections, including those of the respiratory tract, oral mucosa, skin and soft tissues, and abdominal sources were observed in 68.3% patients. Gram-negative bacteria（G^−^）accounted for 79.0% of the isolated bacteria, and gram-positive bacteria（G^+^）accounted for 21.0%. The top five isolated pathogens were Klebsiella pneumoniae（22.5%）, Escherichia coli（20.8%）, Pseudomonas aeruginosa（15.0%）, Enterococcus faecium（5.5%）, and Stenotrophomonas maltophilum（5.0%）. Escherichia coli exhibited a decreasing trend of resistance to quinolones, cephalosporins, and carbapenems. Klebsiella pneumoniae exhibited increasing rates of resistance to quinolones and cephalosporins between 2016 and 2018, but the rated decreased after 2019. The resistance rate to carbapenems exhibited by Pseudomonas aeruginosa was approximately 20%. Carbapenem-resistant strains of Pseudomonas aeruginosa strains were first detected in 2017, with a peak resistance rate of 35.7%, detected in 2019. A 60.0% resistance rate to methicillin was observed in methicillin-resistant coagulase-negative staphylococci（MRCNS）, and one case of linezolid-resistant MRCNS was detected.

**Conclusion:**

Pathogenic bacteria of bacterial bloodstream infections were widely distributed in our center, and precautions are warranted against carbapenem resistant P. aeruginosa and Klebsiella pneumoniae.

血液病患者血流感染发生率为11％～38％，死亡率为6％～36％[1]–[5]。正确的初始经验性抗感染决策对提高患者疗效、降低感染相关死亡率至关重要[6]。CHINET数据显示我国细菌耐药存在地域差异，临床医师合理经验性应用抗生素并做出适宜抗感染决策的重要前提是明确本地区本医院本科室血流感染病原菌分布及耐药情况[7]。本研究对我院2016至2022年血液病患者细菌性血流感染病原学及耐药特点进行回顾性分析和探讨，旨在为临床经验性抗感染治疗决策提供病原学数据依据。

## 病例与方法

1. 病例资料：对我院血液科2016年1月至2022年12月经血培养确诊的细菌性血流感染的血液病患者的一般临床信息、病原菌及药物敏感试验结果进行回顾性收集与分析。粒细胞缺乏（粒缺）的标准为外周血中性粒细胞绝对计数<0.5×10^9^/L或预计48 h后外周血中性粒细胞绝对计数<0.5×10^9^/L[8]。

2. 菌株鉴定及药敏试验：根据常见细菌药物敏感性试验报告规范中国专家共识[9]及2014年美国临床和实验室标准协会（CLSI）标准[10]进行菌株鉴定及药敏试验和结果的判读。

3. 统计学处理：应用SPSS 20.0软件进行统计学分析。组间分类变量的差异性比较采用卡方检验或Fisher精确概率法，以双侧*P*<0.05为差异有统计学意义。应用WHONET5.6软件进行菌株分布及药敏数据分析，计算耐药率。

## 结果

一、患者特征

2016年1月至2022年12月我院血液科共计498例住院患者经血培养确诊为细菌性血流感染，共发生619例次感染，分离出639株细菌。其中18例（6.0％）患者发生复数菌血流感染，分离出38株细菌菌株。77例（15.5％）患者发生多次血流感染，共分离出205株细菌菌种。10.7％（66/619）的患者出现血流动力学改变，75.8％（50/66）处于粒缺期。预后方面，血流感染患者14 d全因死亡率为9.7％，30 d全因死亡率为12.6％。患者临床特征详见表1。

**表1:** 细菌性血流感染患者的临床特征

患者特征	占比[%（阳性例数/总例数）]
年龄≥60岁	44.1（273/619）
原发病​​	
恶性血液病	86.9（538/619）
良性血液病	10.2（63/619）
其他系统恶性肿瘤	1.8（11/619）
感染性疾病及未确诊患者	1.1（7/619）
合并基础疾病	41.6（258/619）
慢性肺部疾病	8.4（52/619）
糖尿病	10.9（68/619）
心脏疾病	2.7（17/619）
肾功能衰竭	1.5（9/619）
自身免疫性疾病	1.5（9/619）
其他系统肿瘤	1.5（9/619）
治疗方案	
化疗/靶向药物治疗	92.1（570/619）
免疫抑制治疗	7.9（49/619）
粒细胞缺乏	76.7（475/619）
导管留置	65.9（408/619）
伴随症状	68.3（423/619）
单部位伴随症状	49.3（305/619）
2个及以上部位伴随症状	19.0（118/619）
呼吸系统	60.6（375/619）
口腔黏膜及皮肤软组织	17.3（107/619）
腹腔	14.5（90/619）
肛周	3.7（23/619）
泌尿系统	2.7（17/619）
导管相关感染	0.6（4/619）
降钙素原升高	32.6（202/619）
长期或反复住院	70.3（435/619）
无菌层流病房住院	6.5（40/619）

二、细菌性血流感染的致病菌分布

血流感染检出的639株细菌中，革兰阴性（G^−^）菌占79.0％（505/639），革兰阳性（G^+^）菌占21.0％（134/639）。前5位病原菌依次为肺炎克雷伯菌（22.5％，144/639）、大肠埃希菌（20.8％，133/639）、铜绿假单胞菌（15.0％，96/639）、屎肠球菌（5.5％，35/639）、嗜麦芽窄食单胞菌（5.0％，32/639）（图1A）。

**图1:**
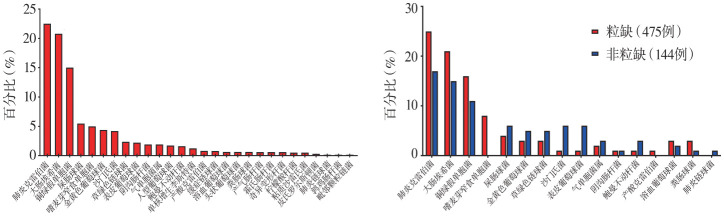
细菌性血流感染患者2016–2022年病原菌分布 **A** 全部患者；**B** 粒细胞缺乏（粒缺）和非粒缺患者病原菌分布比较

18例复数菌血流感染患者中，7例患者同时分离出肺炎克雷伯菌与大肠埃希菌菌株；共分离出细菌38株。其中G^−^菌占78.9％（共30株），G^+^菌占21.1％（共8株），以肺炎克雷伯菌（26.3％，10/38）、大肠埃希菌（26.3％，10/38）为主。

三、粒缺和非粒缺患者病原菌分布比较：粒缺与非粒缺患者均以G^−^菌血流感染为主，粒缺患者G^−^菌比例（85.0％）较非粒缺患者（64.4％）高（*P*<0.001）（图1B）。

四、细菌性血流感染主要病原菌检出率变迁

G^−^杆菌检出率2016年至2020年呈上升趋势，2018年起检出率超过80％，近两年呈下降趋势。其中肺炎克雷伯菌检出率呈上升趋势，2020年检出率最高，近两年逐渐下降，2016年检出率为11.5％，2020年上升至30.9％，2022年下降为20.2％。大肠杆菌和铜绿假单胞菌的检出率保持相对稳定，分别在20％和15％左右波动。嗜麦芽窄食单胞菌的检出率2016年至2018年呈上升趋势，2019年开始呈下降趋势。鲍曼不动杆菌整体呈下降趋势，2019年略有上升，检出率为3.1％。G^+^球菌的检出率2016年至2020年呈下降趋势，2020年以后逐渐上升。其中凝固酶阴性葡萄球菌的检出率下降最为明显，2016年其检出率为15.4％，2019年降至2.1％，近三年呈回升趋势；肠球菌的检出率保持稳定，金黄色葡萄球菌呈下降趋势，但近两年有所回升；链球菌的检出率呈下降趋势（图2）。

**图2:**
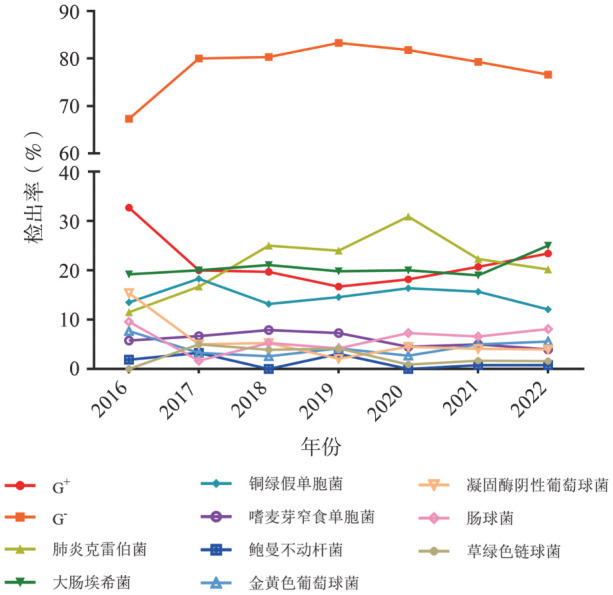
2016–2022年细菌性血流感染主要病原菌检出率变化

五、不同血流感染细菌耐药性分析

1. 主要G^−^杆菌对常用抗菌药物耐药情况：肠杆菌科细菌对头孢哌酮-舒巴坦及喹诺酮类抗生素耐药率较高，对不同头孢菌素的耐药率有所不同（14.1％～46.8％），对β-内酰胺酶抑制剂组合的耐药率为8.9％～16.3％，对替加环素和阿米卡星相对敏感，对卡巴培南类药物的耐药率略高于5％。肠杆菌科细菌中大肠埃希菌的耐药率最高，超过50％的大肠埃希菌对头孢哌酮舒巴坦和左氧氟沙星耐药，大肠埃希菌对第二、三、四代头孢菌素的耐药率（19.3％～42.1％）高于肺炎克雷伯菌（14.1％～26.1％）。铜绿假单胞菌对喹诺酮类抗生素的耐药率为100％，对碳青霉烯类抗生素的耐药率为18.3％，均高于其他G^−^杆菌。主要G^−^杆菌对常用抗生素的耐药率见表2。

**表2:** 2016–2022年主要革兰阴性杆菌对常用抗菌药物的耐药率（％）

抗菌药物	肺炎克雷伯杆菌	大肠杆菌	铜绿假单胞菌	嗜麦芽窄食单胞菌
复方新诺明	32.6	53.2	100.0	0.0
左氧氟沙星	25.0	54.4	10.0	12.0
头孢呋辛	25.0	42.1	/	–
头孢西丁	14.1	27.8	/	–
头孢他啶	17.4	19.3	–	–
头孢曲松	26.1	46.8	–	–
头孢吡肟	20.7	30.4	–	–
阿莫西林/克拉维酸	20.7	24.1	–	–
哌拉西林他唑巴坦	14.1	12.7	10.0	–
头孢哌酮舒巴坦	16.3	8.9	3.3	–
替加环素	9.8	0.0	/	–
厄他培南	6.5	6.3	–	–
亚胺培南	5.4	7.6	18.3	–
阿米卡星	4.3	5.1	0.0	–

**注** –：未进行药敏试验；/：不适用

2. 肺炎克雷伯菌和大肠埃希菌产超广谱β-内酰胺酶（ESBL）和不产ESBL菌株对常用抗菌药物的耐药情况：肺炎克雷伯菌和大肠埃希菌中产ESBL菌株比例分别为24.3％（35/144）和36.8％（49/133）。2016–2022年历年肺炎克雷伯菌产ESBL菌株比例分别为16.7％、20.0％、36.8％、21.7％、25.0％、25.9％和28.0％；历年大肠埃希菌产ESBL菌株比例分别为40.0％、41.7％、31.3％、52.6％、18.2％、43.5％和35.5％。产ESBL菌株对复方新诺明、左氧氟沙星、不同头孢类药物、阿米卡星、β-内酰胺酶抑制剂复方制剂及替加环素的耐药率均高于非产ESBL菌株。其中产ESBL肺炎克雷伯菌耐药率较高，对复方新诺明耐药率超过90％；对不同头孢菌素的耐药率为40.0％～85.7％；对头孢哌酮舒巴坦的耐药率为77.2％；对β-内酰胺酶抑制剂组合的耐药率约为45％；对替加环素的耐药率为37.1％；对碳青霉烯类药物的耐药率为14.3％～20.0％（图3A）。产ESBL大肠杆菌对头孢曲松的耐药率最高，为83.8％，对其他头孢菌素的耐药率为26.5％～77.6％；对左氧氟沙星的耐药率为75.5％；对头孢哌酮-舒巴坦的耐药率为55.1％（图3B）。

**图3:**
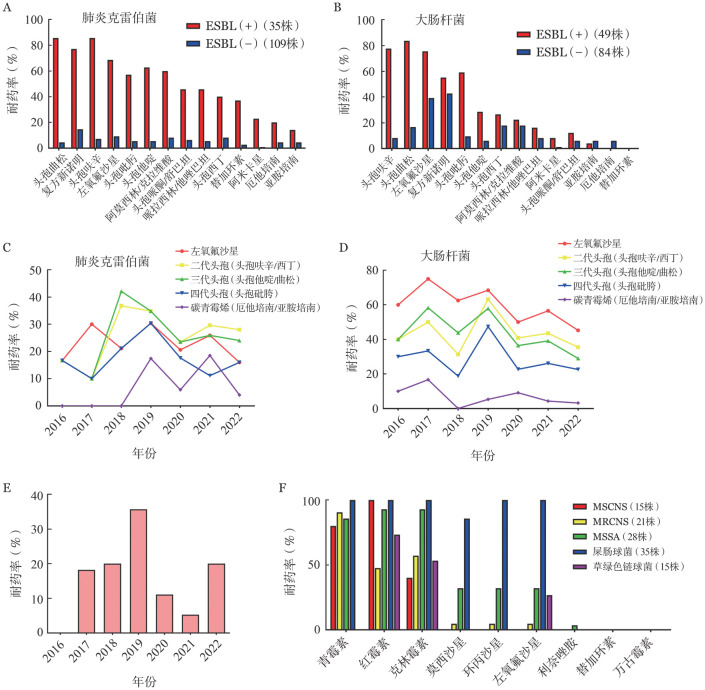
本中心2016–2022年血流感染细菌耐药性分析 **A、B** 产超广谱β-内酰胺酶（ESBL）和不产ESBL肺炎克雷伯菌及大肠杆菌对常用抗生素的耐药模式；**C、D** 肺炎克雷伯菌和大肠杆菌对常用抗生素的耐药模式变化；**E** 铜绿假单胞菌对卡巴培南类药物的耐药模式变化；**F** 主要革兰阳性球菌对常用抗生素的耐药模式 **注** MSCNS：甲氧西林敏感凝固酶阴性葡萄球菌；MRCNS：耐甲氧西林凝固酶阴性葡萄球菌；MSSA：甲氧西林敏感金黄色葡萄球菌

3. 主要G^−^杆菌对常用抗菌药物耐药情况变迁：肺炎克雷伯菌对喹诺酮类、头孢菌素类药物的耐药率2016—2018年呈上升趋势，2019年以后呈下降趋势。大肠埃希菌对喹诺酮类、头孢菌素类、卡巴培南类药物的耐药率均呈下降趋势，2018—2019年耐药率略有波动。耐碳青霉烯类肠杆菌科细菌（CRE）检出率在不同年份出现明显波动，2017年耐碳青霉烯类大肠埃希菌检出率最高（16.7％），2018年未检出。耐碳青霉烯类肺炎克雷伯菌于2019年首次检出。耐碳青霉烯类铜绿假单胞菌于2017年首次检出，2017—2019年其耐药率逐年上升，达到最高峰35.7％。2020年，这一比例下降至11.1％，2022年又上升至20.0％（图3C～E）。

4. 主要G^+^球菌对常用抗菌药物耐药情况：凝固酶阴性葡萄球菌（CNS）中，58.3％（21/36）为耐甲氧西林CNS（MRCNS），MRCNS对青霉素类、大环内酯类、喹诺酮类药物的耐药率均高于甲氧西林敏感CNS（MSCNS），其中检出1例耐利奈唑胺的MRCNS。金黄色葡萄球菌中，均为甲氧西林敏感金黄色葡萄球菌（MSSA），未检出耐甲氧西林金黄色葡萄球菌（MRSA）。屎肠球菌对青霉素类、大环内酯类、喹诺酮类药物的耐药率较高，多重耐药率为100％。在G^+^球菌中，除1例对利奈唑胺耐药的MRCNS病例外，在肠球菌属和链球菌属中均未检测到对利奈唑胺或替加环素耐药的菌株（图3F）。

## 讨论

血流感染是血液病患者的主要并发症，耐药菌的出现是影响患者生命的重要危险因素。如何预防和减少细菌耐药性的产生，需要政府监管、实验室监测、医护人员及公众教育、耐药机制研究、药物研发、临床合理使用抗生素等多方面努力。因此，动态监测本地区病原菌分布及耐药情况，是临床医师制定合理抗菌治疗决策的重要前提。

本中心近7年的回顾性研究显示，细菌性血流感染多于恶性血液病、行化疗及免疫抑制治疗、粒缺及长期反复住院患者中检出。血流感染的原发灶往往隐匿不易寻找。本研究显示68.3％患者在发生血流感染的同时出现其他部位感染的症状，为原发感染灶的可能部位，常见感染部位为呼吸道、口腔黏膜及皮肤软组织及腹腔；其余患者原发感染灶不明。本研究中患者发病时降钙素原（PCT）升高比例占32.6％，考虑与患者粒缺、免疫抑制严重及早期经验性抗细菌治疗相关，且部分病例因脓毒症起病太快而未达到PCT可检测时间窗（多为起病后3～6 h）。文献报道中性粒细胞减少患者合并感染时PCT诱导被抑制，且生成减少，仅为正常的1/2～1/3[11]。PCT可作为感染性疾病诊断、分层、治疗和预后评估的监测指标，但针对血液病患者，不能仅凭PCT水平做出抗感染治疗决策，建议及时复查并监测PCT指标。

本中心细菌性血流感染以G^−^杆菌占主要地位，与国内文献报道一致[12]–[14]。G^−^杆菌以肠杆菌科为主，其中肺炎克雷伯菌、大肠埃希菌位居检出率前2位；其次为非发酵菌，以铜绿假单胞菌及嗜麦芽窄食单胞菌为主。G^+^球菌以葡萄球菌属为主，CNS居首位，其次为屎肠球菌、金黄色葡萄球菌、草绿色链球菌。血流感染分离菌中前5位的病原菌为肺炎克雷伯菌、大肠埃希菌、铜绿假单胞菌、屎肠球菌、嗜麦芽窄食单胞菌。根据《CHINET中国抗菌药物耐药性监测报告（2022年1–12月）》，2022年临床分离菌以G^−^菌为主，占71％，G^+^菌占29％。血培养标本中分离菌中前5位的为大肠埃希菌（20.7％）、肺炎克雷伯菌（15.6％）、表皮葡萄球菌（10.3％）、金黄色葡萄球菌（8.3％）、凝固酶阴性葡萄球菌（6.9％）。我中心大肠杆菌的检出率与CHINET数据一致，肺炎克雷伯菌高于CHINET数据，铜绿假单胞菌明显高于CHINET数据（3.2％），屎肠球菌和嗜麦芽窄食单胞菌稍高于CHINET数据（4.3％和1.2％），这些差异可能与血液病患者免疫系统功能严重受损，临床上采用强化化疗或强免疫抑制治疗有关。

G^−^杆菌中肠杆菌科细菌占我中心血流感染病原菌的68.7％，对头孢哌酮-舒巴坦、喹诺酮类、各代头孢菌素等联合用药的耐药率普遍在20％以上，尤其是产ESBL的菌株耐药明显，此类药物不太适合作为一线经验性抗菌治疗。肠杆菌科细菌对β-内酰胺酶抑制剂联合用药的耐药率仍在10％左右，可作为血液科发热患者初始经验性治疗药物。但值得注意的是，产ESBL的肺炎克雷伯菌对β-内酰胺酶抑制剂联合用药的耐药率已超过1/3，且肺炎克雷伯菌的检出率呈上升趋势。因此，在缺乏明确的微生物学和药敏试验结果的情况下，若将该类药物作为发热和粒细胞减少的血液病患者的初始经验性治疗，应密切监测治疗效果，必要时及时调整抗菌方案。

碳青霉烯类抗生素在治疗多重耐药肠杆菌科细菌感染中具有重要价值[15]–[16]。碳青霉烯类耐药在中国及全球范围内呈上升趋势，成为抗菌治疗领域的全球性挑战[17]–[19]。本中心于2017年首次检出耐碳青霉烯类铜绿假单胞菌菌株，2019年首次检出耐碳青霉烯类肺炎克雷伯菌菌株。肠杆菌科细菌对碳青霉烯类药物的耐药率均超过5％，铜绿假单胞菌对碳青霉烯类药物的耐药率接近20％。2022年CHINET报告中，27 257株铜绿假单胞菌中，对亚胺培南和美罗培南的耐药率分别为17.6％和22.1％，与本中心数据一致。本中心对亚胺培南类药物的耐药情况与国内报道大体相似，在本研究范围内未见明显的耐药率上升趋势。

本研究中G^+^球菌的检出率2016年至2020年已呈下降趋势，2020年以后逐渐上升，考虑G^+^球菌检出率的增加与侵入性操作增加、皮肤黏膜及肺泡上皮细胞损伤、氟喹诺酮类药物预防治疗增加有关。临床应加强对高危患者G^+^球菌特别是耐药菌定植情况定期监测并及时抗球菌治疗。MRSA不同地区感染率不同，从9.8％到57.1％不等[20]–[21]，近年来呈下降趋势，美国CDC报告MRSA血流感染率逐渐下降[22]，CHINET也报告MRSA检出率呈逐年下降，2022年MRSA检出率为28.7％，耐甲氧西林表皮葡萄球菌（MRSE）为82.2％，其他MRCNS为77.6％。在我们的研究中，血培养标本中未检测到MRSA。MRCNS占比为58.3％，低于国内及国际报道的耐药率[23]。本中心除1例对利奈唑胺耐药的MRCNS病例外，在葡萄球菌、肠球菌及链球菌中均未检测到对利奈唑胺、万古霉素及替加环素耐药的菌株，该耐药率低于国内（2.2％）及国际（27.6％）的万古霉素耐药率[24]–[25]。因此，万古霉素及利奈唑胺仍可作为治疗G^+^球菌感染的首选药物。

综上所述，本中心血液病患者血流感染以G^−^杆菌为主，致病菌以大肠埃希菌、肺炎克雷伯菌、铜绿假单胞菌为主，屎肠球菌、嗜麦芽窄食单胞菌的检出率明显高于全国。大肠埃希菌对喹诺酮类、头孢菌素类、碳青霉烯类药物的耐药率呈下降趋势，肺炎克雷伯菌对喹诺酮类、头孢菌素类药物的耐药率呈上升趋势。CRE的检出率每年波动较大，耐碳青霉烯类的铜绿假单胞菌也呈逐年上升趋势。
